# Using Delphi methodology in the development of a new patient‐reported outcome measure for stroke survivors with visual impairment

**DOI:** 10.1002/brb3.898

**Published:** 2018-01-17

**Authors:** Lauren R. Hepworth, Fiona J. Rowe

**Affiliations:** ^1^ Department of Health Services Research University of Liverpool Liverpool UK

**Keywords:** Delphi process, patient‐reported outcome measure development, quality of life, stroke, visual impairment

## Abstract

**Introduction:**

The aim of this study was to ascertain what items stroke survivors and stroke care professionals think are important when assessing quality of life for stroke survivors with visual impairment for inclusion in the new patient‐reported outcome measure.

**Methods:**

A reactive Delphi process was used in a three‐round electronic‐based survey. The items presented consisted of 62 items originally sourced from a systematic review of existing vision‐related quality of life instruments and stroke survivor interviews, reduced and refined following a ranking exercise and pilot with stroke survivors with visual impairment. Stakeholders (stroke survivors/clinicians) were invited to take part in the process. A consensus definition of ≥70% was decided a priori. Participants were asked to rank importance on a 9‐point scale and categorize the items by relevance to types of visual impairment following stroke or not relevant. Analysis of consensus, stability, and agreement was conducted.

**Results:**

In total, 113 participants registered for the Delphi survey of which 47 (41.6%) completed all three rounds. Response rates to the three rounds were 78/113 (69.0%), 61/76 (81.3%), and 49/64 (76.6%), respectively. The participants included orthoptists (45.4%), occupational therapists (44.3%), and stroke survivors (10.3%). Consensus was reached on 56.5% of items in the three‐round process, all for inclusion. A consensus was reached for 83.8% in the categorization of items. The majority (82.6%) of consensus were for relevant to ‘all visual impairment following stroke’; two items were deemed ‘not relevant’.

**Conclusion:**

The lack of item reduction achieved by this Delphi process highlights the need for additional methods of item reduction in the development of a new PROM for visual impairment following stroke. These results will be considered alongside Rasch analysis to achieve further item reduction. However, the Delphi survey remains important as it provides clinical and patient insight into each item rather than purely relying on the psychometric data.

## 1 INTRODUCTION

The point prevalence of visual impairment in stroke survivors has been reported as 72% (Hepworth et al., 2015; Rowe, Hepworth, Hanna, & Howard, 2016). Visual impairment as a result of stroke takes different forms across four main categories: visual field loss, ocular motility defects, reduced visual acuity, and visual perception problems (Hepworth et al., 2016a). These impairments have the potential to affect an individual's ability to perform activities of daily living (ADLs), for example, self‐care, mobility, and socializing (Hepworth & Rowe, 2016a). An individual with visual impairment may have reduced level of independence. A combination of limitations has the potential to impact on an individual's mood and motivation. These sequelae have been reported in populations with visual impairment (Chia et al., 2004; McBain et al., 2014; Tsai et al., 2003; Wang, Chan, & Chi, 2014).

A systematic narrative review of existing instruments for measuring vision‐related quality of life demonstrated a need for the development of a new patient‐reported outcome measure (PROM) with a specific focus on the impact of the wide variety of visual impairments following stroke (Hepworth et al., 2015). It was considered important that development of the new PROM was carried out in collaboration with stroke survivors with visual impairment. The development method for the new instrument adopted two methods of instrument development, Rasch analysis and a Delphi process, providing both psychometric and experiential knowledge to inform each other.

In order to ascertain what items stroke survivors and stroke care professionals think are important when assessing quality of life for stroke survivors with visual impairment and for inclusion in the new patient‐reported outcome measure, we sought in this study to identify:

1. Which items were important in the assessment of quality of life with visual impairment following stroke to aid development of a new patient‐reported outcome measure,
2. A ‘hub’ core item set in addition to spoke items for specific visual impairment following stroke, for example, visual field loss, ocular motility defects, visual perception problems.

## 2 METHOD

A reactive Delphi process was used in a three‐round electronic‐based survey. The survey involved two parts. The first asked participants to judge the importance of 62 items on a 9‐point scale, from 1 ‘not important’ to 9 ‘critical’. The second asked participants to categorize if the same 62 items were relevant to ‘all types of visual impairment following stroke’ or to specific taxonomies (‘reduced central vision’, ‘visual field loss’, ‘ocular motility defects’ or ‘perceptual problems’) or were considered ‘not relevant to visual impairment following stroke’.

Sixty‐two items were presented in this Delphi survey. These 62 items were selected from 102 items, which were developed from the coded themes of items originally sourced from a systematic review of 34 existing vision‐related quality of life instruments (Hepworth et al., 2015). The 102 items were cross‐checked with the interview transcripts of 35 stroke survivors—no new items were required (Rowe, 2017). All items were unified and worded to allow the extraction of the specific impact of visual impairment following stroke from the impact of other sequelae of stroke. They were then ranked for importance by 60 clinicians and 61 stroke survivors and piloted with 37 stroke survivors with visual impairment. The items which were not relevant or overlapping were removed, and some reworded resulting in the 62 items which required further evaluation (Hepworth & Rowe, 2016b).

### 2.1 Consensus

The end point used for this study was the number of rounds, set at three, to limit attrition of participants (Cantrill, Sibbald, & Buetow, 1996). Consensus was defined ‘a priori’. If ≥70% of participants scored the item as ‘critical’ (options 7–9) and <15% of participants scored the item as ‘not important’ (options 1–3), the item was prioritized. Items were considered for removal if ≥70% of participants scored the item as ‘not important’ (options 1–3) and <15% of participants scored the item as ‘critical’ (options 7–9). All other scoring patterns were taken to indicate nonconsensus (Harman et al., 2013).

In part two, consensus could be achieved if 70% of participants allocated an item to either ‘relevant to all visual impairment following stroke’ or ‘not relevant to visual impairment following stroke’. In cases where an item might be relevant to more than one taxonomy (reduced central vision, visual field loss, ocular motility defect, and visual perception), if the total across three or less of the categories reached 70%, consensus was deemed to have been achieved. Fewer than 15% must have chosen the opposing standpoint ‘not relevant to all visual impairment following stroke’ or ‘relevant to all visual impairment following stroke’.

### 2.2 Participants

Stroke survivors and clinicians with knowledge of visual impairment following stroke were targeted: stroke survivors with visual impairment resulting from stroke, orthoptists and occupational therapists involved in stroke care. An advertisement outlining the project was used to identify participants. Potential participants emailed the research team if expressing interest.

### 2.3 Survey rounds

All volunteers were emailed a link to the survey. The opening page of the survey acted as both the participant information sheet and consent form. The order in which the items were presented to each participant was randomized in round one. Nonresponders or partial completers in each round were sent two reminder emails, which included an option to withdraw from the study. Participants who completed the previous round were sent the link to the next round survey along with their individual responses. The order of the items was not randomized from round two onwards, allowing the individual responses to be presented in the same order as the items in the survey. Items were not removed between rounds; therefore, the number of items remained the same in each round.

### 2.4 Data analysis

Group feedback was prepared using histograms to show the distribution of responses as one group. Individual response sheets were also prepared.

Part one of the survey was analyzed using the Holey and colleagues method of assessing consensus and stability (Holey, Feeley, Dixon, & Whittaker, 2007):

1. Percentage response rates.
2. Level of agreement in percentage terms for each item to allow for differing response rates.
3. Median and range
4. Mean and standard deviation, along with rank of importance for each item
5. Weighted Kappa (K) values—assessing chance‐eliminated agreement between rounds one and two, rounds two and three, and rounds one and three.

The categorical data of part two were analyzed using percentage response rates, against the consensus definition.

## 3 RESULTS

### 3.1 Response rate

**Figure 1:**
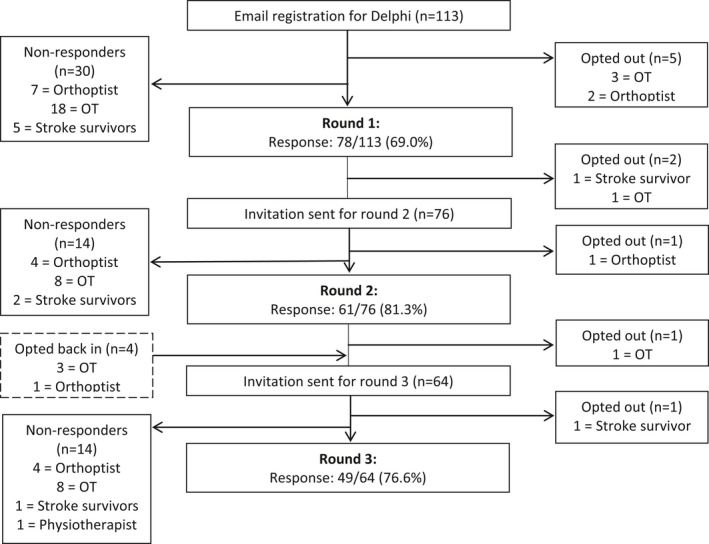
Flowchart showing responses to Delphi survey, rounds 1–3

In total, there were 113 expressions of interest registered for participating in the Delphi survey. Response rates to the three rounds were 78 of 113 (69.0%), 61 of 76 (81.3%), and 49 of 64 (76.6%), respectively (Figure 1). Of the original emails of interest, 47 participants (41.6%) participated in all three rounds and 30 (26.5%) did not participate in any of the rounds.

### 3.2 Demographics

All demographics collected from the first round and tracked through the process are outlined in Table 1. Participants were predominantly clinicians (87.2%–89.8%). The clinical professionals were an almost equal balance between occupational therapists (OTs) (51.5%–45.5%) and orthoptists (47.0%–54.5%). A small group of stroke survivors (12.8%–10.2%) participated in the survey. The majority of the stroke survivors had visual field loss; however, two other major visual impairment categories (ocular motility defects and visual perception problems) were represented. The participants were predominantly female (88.5%–91.8%).

**Table 1:** Demographics of participants to Delphi survey, rounds 1–3

	Round 1, *n* (%)	Round 2, *n* (%)	Round 3, *n* (%)
**All participants**	78	61	49
Male	9 (11.5)	5 (8.2)	4 (8.2)
Female	69 (88.5)	56 (91.8)	45 (91.8)
18–24 years	1 (1.3)	1 (1.6)	0 (0.0)
25–34 years	16 (20.5)	13 (21.3)	11 (22.4)
35–44 years	26 (33.3)	18 (29.5)	14 (28.6)
45–54 years	26 (33.3)	21 (34.4)	19 (38.8)
55–64 years	8 (10.3)	7 (11.5)	4 (8.2)
65–74 years	0 (0.0)	0 (0.0)	0 (0.0)
75–84 years	1 (1.3)	1 (1.6)	1 (2.0)
85 years and older	0 (0.0)	0 (0.0)	0 (0.0)
**Stroke survivors**	10 (12.8)	7 (11.5)	5 (10.2)
Visual field loss	7 (70.0)	4 (57.1)	3 (60.0)
Visual perception	1 (10.0)	1 (14.3)	1 (20.0)
Ocular motility defect	2 (20.0)	2 (28.6)	1 (20.0)
**Clinicians**	68 (87.2)	54 (88.5)	44 (89.8)
Occupational therapists	35 (51.5)	26 (48.1)	20 (45.5)
Orthoptists	32 (47.0)	27 (50.0)	24 (54.5)
Physiotherapists	1 (1.5)	1 (1.9)	0 (0.0)

Additional demographics were collected in the third round. These demonstrated that the clinicians completing the third round were highly experienced in both number of years and types of setting. Fifty percent (*n* = 22) of clinicians had more than 10 years’ experience working with stroke survivors, and only one participant had less than 1 years’ experience. The cohort also worked across the whole care pathway from acute stroke units to outpatient appointments and community home visits. Forty‐one percent (*n* = 18) of clinicians worked in two or more of these settings, with nine percent (*n* = 4) covering four settings. The stroke survivors completing the third round were also highly experienced; two had lived with their stroke‐related visual impairment for over 10 years and three for between three and seven years. The geographical spread of responses was wide and included England, Ireland, Scotland, Wales, and Jersey.

### 3.3 Consensus and stability evolution

#### 3.3.1 Importance

**Figure 2:**
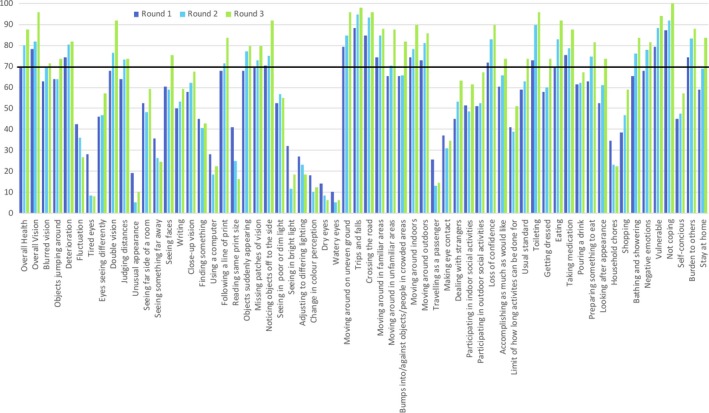
Percentage responses for each item to the “critical” (7–9) option. The line at 70% indicates consensus was achieved, no items which reached this point had ≥15% in the opposing “not important” (1–3) category

Consensus was reached on 55% (*n* = 34) of items across the three‐round process for part one, all of which were deemed ‘critical’ and therefore were for inclusion. The percentage response to the ‘critical’ (7–9) category across all three rounds for each item is outlined in Figure 2. Of the items achieving consensus, 15 were reached in the first round, a further 11 in the second round and a further nine in the third round. The rank order achieved by each item and those achieving consensus at the end of round 3 is shown in Table 2. Seventy‐six percent of the items achieving consensus were from four categories: ‘moving around’ (23.5%), ‘independent living’ (20.6%), ‘well‐being’ (17.6%), and ‘general vision’ (14.7%). The remaining eight items achieving consensus were from four categories: ‘peripheral vision’, ‘reading’, ‘near vision’, and ‘role limitation’, in addition to the two general items ‘overall health’ and ‘overall vision’. Four items were ranked higher than others achieving consensus. This was the result of more participants choosing either higher ‘eight’ or ‘nine’ categories for those items with insufficient responses within the ‘critical’ category overall.

**Table 2:** Items mean rank position after completion of round 3

Rank	Item	Rank	Item
**=1**	Toileting*	**=32**	Objects jumping around*
**=1**	Not coping*	**=32**	Getting dressed*
**3**	Trips and falls*	34	Pouring a drink
**4**	Overall vision*	**35**	Seeing faces*
**5**	Vulnerable*	=36	Participating in indoor social activities
**6**	Crossing the road*	=36	Participating in outdoor social activities
**7**	Double vision*	**=36**	Looking after appearance*
**8**	Burden to others*	39	Dealing with strangers
**9**	Taking medication*	40	Usual standard
**10**	Loss of confidence*	41	Seeing far side of a room
**11**	Moving around indoors*	42	Shopping
**12**	Negative emotions*	=43	Writing
**13**	Moving around on uneven ground*	=43	Limit of how long activities can be done for
**14**	Eating*	=45	Eyes seeing differently
**15**	Deterioration of vision*	=45	Self‐conscious
**16**	Moving around in familiar areas*	47	Seeing in poor or dim light
**17**	Noticing objects off to the side*	48	Finding something
**18**	Bumps into or against objects or people in crowded areas*	49	Using a computer
**19**	Stay at home*	50	Making eye contact
**20**	Preparing something to eat*	51	Seeing in bright light
**=21**	Moving around in unfamiliar areas*	=52	Fluctuation
**=21**	Moving around outdoors*	=52	Household chores
**23**	Bathing or showering*	54	Seeing something far away
**24**	Judging distances*	55	Adjusting to differing lighting
**=25**	Following a line of print*	56	Reading same print size
**=25**	Missing patches of vision*	57	Travelling as a passenger
**27**	Overall health*	58	Tired eyes
=28	Close‐up vision	59	Unusual appearance
**=28**	Accomplishing as much as would like*	60	Change in colour perception
**=30**	Blurred vision*	61	Dry eyes
**=30**	Objects suddenly appearing*	62	Watery eyes

The * identifies the items which reached consensus. The = sign next to the rank shows that those items are of equal rank.

#### 3.3.2 Categorization

Consensus was reached for 84% (*n* = 52) of items across the three‐round process for part two. Of these 21 were reached in the first round, a further 22 in the second round, and a further nine in the third round. However, of the items which reached consensus in the second round, five subsequently lost this in the third round. The majority (83%, *n* = 43) of the consensus were relevant to ‘all visual impairment following stroke’. Of the remainder achieving consensus, two were for a single category, four were across two categories, one was across three categories, and two were deemed ‘not relevant to visual impairment following stroke’.

### 3.4 Agreement

The level of within‐participant agreement was investigated between the rounds of the survey. The greatest amount of agreement was found between the second and third rounds, with 59.7% (*n* = 37) of items having an increased level of agreement from that between the first and second rounds. The majority of items between rounds two and three had either moderate (Kappa 0.41–0.6) or substantial (Kappa 0.61–0.8) agreement: 40.3% (*n* = 25) and 46.8% (*n* = 29), respectively. Three items (‘overall vision’, ‘making eye contact’, and ‘not coping’) had fair (Kappa 0.21–0.4) agreement between rounds two and three. Five items had almost perfect (Kappa 0.81–1.0) agreement between rounds two and three: ‘blurred vision’, ‘fluctuation’, ‘adjusting to differing lighting’, ‘negative emotions’, and ‘vulnerable’. These five items had a spread of if and when consensus was achieved: no consensus was achieved for two items and consensus was achieved for: one in the first round, one in the second round, and one in the third round.

The majority of items between rounds one and two also had either moderate 56.5% (*n* = 35) or substantial agreement 33.9% (*n* = 21). The remaining six items had fair agreement.

The greatest amount of disagreement was found between the first and third rounds, with 83.9% (*n* = 52) of items showing the lowest level of agreement compared that between rounds one and two and rounds two and three. The majority of items between rounds one and three had either fair 48.4% (*n* = 30) or moderate agreement 37.1% (*n* = 23). Three items demonstrated poor agreement (Kappa 0.0–0.2), all between the first and third rounds: ‘making eye contact’, ‘toileting’, and ‘stay at home’, The ‘toileting’ and ‘stay at home’ items achieved consensus, in the first and third round, respectively, whereas ‘making eye contact’ did not achieve consensus within the three‐round process.

## 4 DISCUSSION

No items were removed by consensus of being deemed unimportant. However, the decision to remove the ‘dry eyes’ and ‘watery eyes’ items was based on the consensus decision that these items were ‘not relevant to visual impairment following stroke’.

Considering the items achieving consensus within the three rounds of this Delphi survey, 34 items under eight categories (‘general vision’, ‘independent living’, ‘moving around’, ‘near vision’, ‘overall health’, ‘peripheral vision’, ‘reading’, and ‘well‐being’) were considered important in the assessment of quality of life for stroke survivors with visual impairment. The categories removed were ‘distance vision’, ‘light’, ‘discomfort’, and ‘socializing’.

For these 34 items to be covered by existing questionnaires would require the use of multiple instruments, with the potential for item duplication and a high task burden. Some of the existing questionnaire which may be required have already been identified as not suitable to be used for stroke survivors due to question phasing issues (Hepworth et al., 2015). It would not be possible to cover all items with existing instruments due to changes made to the items during the pilot development work.

The National Eye Institute Visual Function Questionnaire 25 (NEI VFQ‐25) used by five studies to measure impact on quality of life had six common subscales with reduced scores for stroke survivors with visual field loss compared to healthy individuals. These subscales included general health, general vision, near activities, vision‐specific mental health, driving, and peripheral vision (Chen et al., 2009; Gall, Franke, & Sabel, 2010; Gall, Lucklum, Sabel, & Franke, 2009; Gall et al., 2008; Papageorgiou et al., 2007). All the above categories had at least one item achieving consensus within the Delphi survey, with the exception of driving which did not feature in the survey. The NEI VFQ‐25 was also used in one study which included a study population with reduced visual acuity in addition to visual field loss. As a consequence, the list of subcategories with reduced scores was extended to also include distance vision, social functioning, role difficulties, and dependency (Gall et al., 2010). Items which related to dependency or independent living featured heavily in the items which achieved consensus in the Delphi survey.

**Figure 3:**
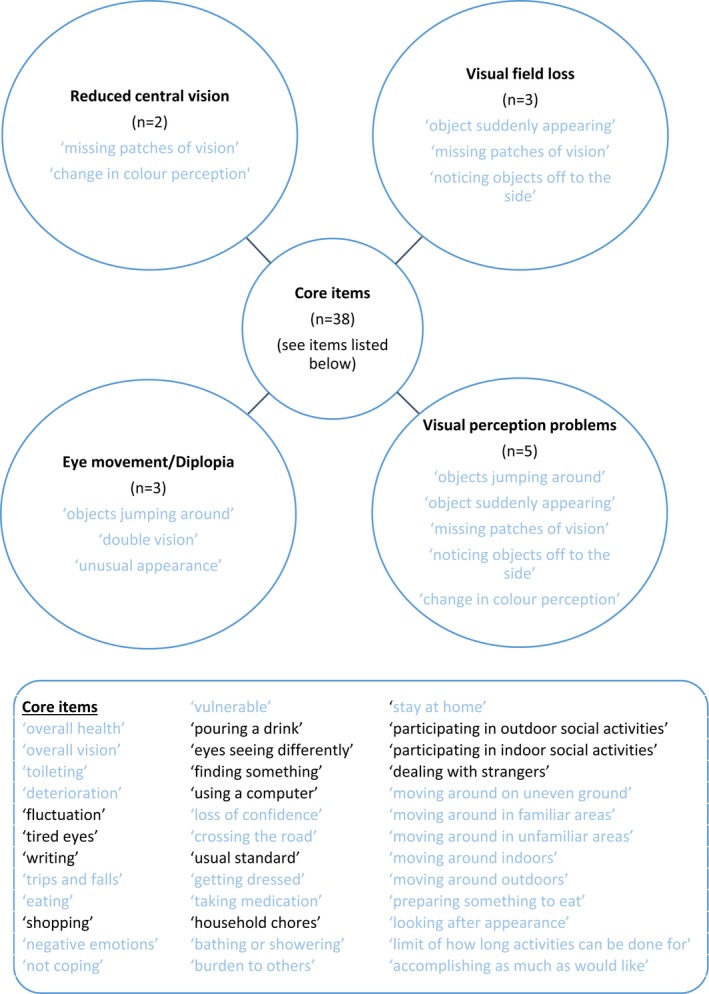
Hub and spoke model of questionnaire of items with consensus from the Delphi survey. The items listed in blue are those that reached consensus in part 1 in terms of importance

One of the aims of this survey was to identify items which could be used to form a hub and spoke model. However, the set of items which were considered relevant to ‘all visual impairment following stroke’ based on this analysis would still result in a large number of core items (*n* = 38) with few additional spoke items (*n* = 13), shown in Figure 3.

The response rate for the survey (41.6%) was good compared to average figures reported by survey companies (24.8%) (FluidSurveys Team, 2014). Even with a dropout rate in the second (21.8%) and third (23.5%) round, the response rate remained good at 62.8% in the final round. A dropout rate of any size carries the risk of nonresponder bias. Those who took the decision not to continue participating in the process may have had different views to those completing all three rounds of the survey (Greatorex & Dexter, 2000). Various steps were taken within the method of this survey delivery to minimize attrition. These included personalizing messages, which have been shown to significantly increase response rate as well as the number completing the task (Heerwegh, 2005; Keeney, Hasson, & McKenna, 2006; Sánchez‐Fernández, Muñoz‐Leiva, & Montoro‐Ríos, 2012). Up to two email reminders were sent with the final reminder including the closing date of the survey. In previous studies, it is has been shown the combination of personalization and reminders creates the largest effect on retention (Sánchez‐Fernández et al., 2012).

Despite these steps, the extent of the survey remained lengthy throughout the three rounds. No items were dropped when they reached consensus, to enable a measure of agreement (weighted Kappa) between the rounds. It is known that the time burden of the survey resulted in attrition of some participants (Keeney, Hasson, & McKenna, 2011). Within all emails participants were given the opportunity to withdraw and were asked to provide a reason for doing so, to enable a clearer understanding of the final round participants. However, in this survey, a large proportion of those that dropped out did so by not responding. A benefit of having level of agreement data is it allows analysis of the quality of the group's decision (Greatorex & Dexter, 2000).

A limitation of using a web‐based survey was that not all stroke survivors with visual impairment have access to or are able to use a computer. This may have prevented some stroke survivors from participating and may have resulted in a younger group of stroke survivors participating. Initially, 15 stroke survivors registered an interest in the study; ten completed the first round which dropped to five by the third round—which we recognize as a further limitation of this study. To counter this, further stages of validation and implementation of this PROM will engage with stroke survivors and their carers to ensure their continued input to this process, just as we have sought from the outset of the development of this PROM.

Development involving patients and clinicians is deemed a key part of creating a high‐quality instrument (Khadka, McAlinden, & Pesudovs, 2013). Building this collaboration into the development of a new instrument improves the potential quality of the final product. The Delphi survey alone also allows an insight into what stroke survivors and clinicians consider important issues impacting quality of life following a stroke with associated visual impairment. However, it appears insufficient to be the sole method to take forward development of a new instrument. Additional methods to be used include consensus meetings and Rasch analysis. The combination of these methods serves to enhance content validity and establish good psychometrics.

## 5 CONCLUSION

The lack of item reduction achieved by this Delphi process highlights the need for additional methods of item reduction in the development of a new PROM for visual impairment following stroke. The results of this Delphi survey will be considered alongside Rasch analysis to achieve further item reduction. However, the Delphi survey remains important as it provides the clinical and patient insight into each item rather than purely relying on the psychometric data provided by Rasch analysis.
